# Flow cytometry analysis: A quantitative method for collagen VI deficiency screening

**DOI:** 10.1016/j.nmd.2011.08.006

**Published:** 2012-02

**Authors:** J. Kim, C. Jimenez-Mallebrera, A.R. Foley, M. Fernandez-Fuente, S.C. Brown, S. Torelli, L. Feng, C.A. Sewry, F. Muntoni

**Affiliations:** a Dubowitz Neuromuscular Centre, University College London Institute of Child Health, London, UK; b Department of Veterinary Basic Science, Royal Veterinary College, London, UK; c Wolfson Centre for Inherited Neuromuscular Disease, RJAH Orthopaedic Hospital, Oswestry, UK; d Neuromuscular Unit, Neuropaediatrics Department, Hospital Sant Joan de Deu, Barcelona, Spain

**Keywords:** Ullrich congenital muscular dystrophy (UCMD), Bethlem myopathy (BM), Collagen VI, Diagnosis, Flow cytometry

## Abstract

Mutations in *COL6A1*, *COL6A2* and *COL6A3* genes result in collagen VI myopathies: Ullrich congenital muscular dystrophy (UCMD), Bethlem myopathy (BM) and intermediate phenotypes. At present, none of the existing diagnostic techniques for evaluating collagen VI expression is quantitative, and the detection of subtle changes in collagen VI expression remains challenging.

We investigated flow cytometry analysis as a means of quantitatively measuring collagen VI in primary fibroblasts and compared this method with the standard method of fibroblast collagen VI immunohistochemical analysis. Eight UCMD and five BM molecularly confirmed patients were studied and compared to five controls.

Flow cytometry analysis consistently detected a reduction of collagen VI of at least 60% in all UCMD cases. In BM cases the levels of collagen VI were variable but on average 20% less than controls.

Flow cytometry analysis provides an alternative method for screening for collagen VI deficiency at the protein level in a quantitative, time and cost-effective manner.

## 1 Introduction

Collagen VI is an ubiquitous extracellular matrix protein composed of three different peptide chains, α1(VI), α2(VI) and α3(VI), encoded by the *COL6A1*, *COL6A2* and *COL6A3* genes. After several intracellular assembly steps, tetramers are secreted from the cell to the extracellular matrix where they assemble into beaded microfilaments [1,2]. Further collagen VI chains, α4(VI), α5(VI) and α6(VI) have been identified and contribute to the complexity of collagen VI function in the extracellular matrix [3,4].

Mutations in any of the three collagen 6A genes *COL6A1*, *COL6A2* and *COL6A3* result in Ullrich congenital muscular dystrophy (UCMD), Bethlem myopathy (BM) or phenotypes intermediate between UCMD and BM, characterised by a combination of distal laxity, proximal muscle weakness and joint contractures [5,6]. It was initially thought that autosomal dominant mutations in the collagen VI genes were associated with the milder BM variant whilst the more severe variant of UCMD was attributed to autosomal recessive inheritance [7]. More recently, however, it has become clear that a significant proportion of UCMD patients carry *de novo* dominant mutations and some BM cases with recessive mutations have been described [8–11]. Furthermore, a recent paper suggested that myosclerosis myopathy should be regarded as a collagen VI related disorder. Two myosclerosis myopathy patients (siblings from a consanguineous family) presented with collagen VI defects and broaden the phenotype and complexity of collagen VI disorders [12].

Collagen VI expression can be analysed by labelling muscle sections with antibodies to collagen VI (alone or in combination with antibodies against a marker of the basal lamina such as perlecan or collagen V) [1,13]. This analysis is dependent on the availability of a muscle biopsy in which the integrity of the basal lamina allows this detailed study. *In vitro* analysis of collagen VI production can also be studied in cultures of fibroblasts, which are a source of collagen VI production in skin and muscle [14]. The conventional analysis of collagen VI immunohistochemistry in skin-derived fibroblasts is, however, both a laborious and non-quantitative technique which has limitations when applied in diagnostic clinical settings [1].

Although the gold standard for the diagnosis of collagen VI myopathy is sequencing of the *COL6A1*, *COL6A2* and *COL6A3* genes, this technique is complicated by both the large size of the three genes (107 coding exons in total) and the high frequency of polymorphisms [8]. It is desirable, therefore, to have a robust system in place to ensure that patients studied with genetic techniques have evidence of collagen VI deficiency.

Flow cytometry analysis has been shown to be effective in various research applications. In the dysferlinopathies, for example, the use of whole blood flow cytometry has been demonstrated to be a reliable, alternative diagnostic tool for this muscular dystrophy which, like the collagen VI myopathies, requires a diagnostic strategy preceding genetic sequencing [15]. The use of flow cytometry analysis has further clinical applications in leukaemia, lymphoma and HIV [16]. Specifically, the superiority of the flow cytometry technique in immunophenotyping of lymphomas in lymph node biopsies has been illustrated, with flow cytometry significantly more sensitive than frozen-section immunoperoxidase for demonstrating light-chain restriction [17]. Flow cytometry analysis also has also had important applications in skeletal muscle research, including its use in sorting satellite cells from muscle fibres [18] as well as muscle specific proteins from murine muscle fibres [19].

In this study we have investigated the potential value of flow cytometry analysis in assessing collagen VI expression in skin-derived fibroblasts, as a screening method prior to undertaking *COL6A1*, *COL6A2* and *COL6A3* gene sequencing.

## 2 Methods

### 2.1 Patients

A total of 13 collagen VI myopathy patients were included in this study. Eight patients have a diagnosis of Ullrich congenital muscular dystrophy with confirmed mutations in *COL6A1* (UCMD patients 7, 12 and 13), *COL6A2* (UCMD patients 8, 10 and 11) and *COL6A3* (UCMD patients 6 and 9) genes. All of these UCMD cases have been demonstrated to have abnormal collagen VI expression by immunohistochemical staining of skin-derived fibroblast cultures Table 1 [1] [20]. The remaining five patients have been diagnosed with Bethlem myopathy with confirmed mutations in *COL6A1*, including a father–son pair (BM 4 and 5). *COL6A1*, *COL6A2* and *COL6A3* mutations in three patients were identified using single condition amplification/internal primer (SCAIP) sequencing. These mutations have been previously reported as part of a collaborative study [8] (Table 1).

### 2.2 Skin biopsy and fibroblast culture

Skin biopsies were taken from patients after obtaining informed patient consent. Control skin biopsy samples were obtained from paediatric patients (undergoing surgical procedures) with no known neuromuscular disease as well as from adults with no known neuromuscular disease following written informed research consent. Fibroblasts were grown from skin explants and cultured in Dulbecco’s modified Eagles medium (Invitrogen) supplemented with 20% foetal bovine serum (FBS, PAA), 1% l-glutamine (Sigma) and 1% penicillin, streptomycin and neomycin (Sigma). Cells were cultured at 37 °C in 5% CO_2_.

### 2.3 Collagen VI immunostaining of fibroblast cultures

10^5^ cells were seeded onto clean coverslips coated with fibrillar collagen I solution (Purecol 5409, Nutacon). When confluent, the medium was changed to contain 50 μg/ml of l-ascorbic acid phosphate to allow for the correct post-translational modification and secretion of collagen molecules. The medium was subsequently changed and ascorbic acid added every 2 days for 7 days’ duration. Cells were then fixed with fresh 2% paraformaldehyde (pH adjusted to 7.4 ± 0.2) for 10 min. An aliquot of cells was permeabilised with Triton X-100 (VWR, 0.05% in PBS) for 3 min. All cells were immunolabelled with a monoclonal primary antibody against collagen VI (MAB1944, Millipore) diluted in PBS/Triton or PBS alone for 1 h (1/500) at room temperature. Coverslips were washed 3 times with PBS (with or without Triton), and anti-mouse biotinylated secondary antibody was applied for 30 min (1/200, Amersham) at room temperature which was followed by washing with streptavidin-conjugated Alexa 594 (1/1000, Invitrogen) for 15 min at room temperature. Nuclei were stained with Hoescht 33342 (1/2000, Molecular Probes) for 5 min, and preparations were examined under a Leica DMR epifluorescent microscope linked to Metamorph software (Universal Imaging).

### 2.4 Flow cytometry analysis method: Immunostaining of fibroblasts

Primary skin fibroblast cultures (standardised for passage number and at a density of 8 × 10^5^) were grown in 75 cm^2^ tissue culture flasks (BD). When cells were confluent, 50 μg/ml of l-ascorbic acid phosphate was added, and cells were further incubated for 24 h. This time point was chosen because in previous experiments we had shown that collagen VI was already deposited in the extracellular matrix by this time point, and the differences between control and patient cultures could be readily detected (data not shown). Cells were harvested with a non-enzymatic cell dissociation solution (Sigma) and fixed with 2% paraformaldehyde for 10 min on ice. Afterwards, cells were washed with PBS (Mg^2+^ and Ca^2+^ free, Invitrogen) containing 0.1% FBS and centrifuged. Pellets were re-suspended and incubated with a monoclonal primary antibody against collagen type VI (Millipore, MAB1944, 1/250) in PBS/0.1% FBS or PBS/ 0.05% FBS and Tween 20 (for permeabilisation) for 1 h. For a negative control, cells were incubated without primary antibody on ice. Cells were washed twice with or without 0.05% Tween 20 and then spun down at 3000*g* for 3 min. Rabbit anti-mouse IgG conjugated to R.Phycoerythrin (RPE, Star12A, SeroTec) was diluted (1/20) with either PBS/0.1% FBS or PBS/0.1% FBS and 0.05% Tween 20 for 20 min on ice. Cells were then washed twice with PBS (with or without Tween 20, as appropriate) and centrifuged at 3000*g* for 3 min. Finally, cells were re-suspended in PBS/0.1% FBS and filtered through a 0.7 μm strainer to ensure cells were separated individually. Cells were then processed for flow cytometry and run on a Cyan ADP (Beckman Coulter, California, USA) fitted with a 488 nm laser and a 633 nm red diode. Data analysis was done on Summit (Beckman Coulter, California, USA) or Flowjo (TreeStar, Oregon, USA) software. A total of 15,000 cells were analysed and gated using the two-parameter analysis side light scatter (SSC; linear scale) on the *y*-axis and fluorescence intensity (PE-collagen VI; log scale) on the *x*-axis. Side scatter (SS) is related to the cellular cytoplasmatic granularity and PE fluorescence is collected with a 575 nm ± 12.5 band-pass filter (FL2 detector). A negative control (where primary antibody is omitted) was used to set up collagen VI positive immunolabelling gate and the same gate was applied to all samples.

### 2.5 Statistical analysis

Flow cytometry results from non-permeabilised and permeabilised cells are expressed as mean ± standard deviation. Multiple regression analysis was performed (R, version 2.12.0) to assess differences among the groups.

## 3 Results

The clinical features of all patients included in this study are summarised in Table 1. Fibroblast cell cultures from all 13 patients had been tested previously and demonstrated abnormal organisation of the collagen VI network [1] (Fig. 1a). Regarding UCMD and BM patients for whom muscle biopsy specimens were available, we observed reduced collagen VI immunolabelling in the basal lamina, as detailed in Table 1.

Prior to the flow cytometry analysis, fibroblasts were examined under a Leica DMR epifluorescent microscope to confirm that collagen VI was expressed on the cell surface (data not shown). For flow cytometry analysis, cells were divided into three groups: a control group, a BM group (BM1–BM5) and an UCMD group (UCMD6–UCMD13) (Fig. 1b). BM and UCMD groups were compared to the control group (non-permeabilised cells) (Fig. 2a).

The five control cell lines demonstrated a mean collagen VI surface expression of 65.4 ± 10.7. When the control cells were permeabilised, all demonstrated > 90% collagen VI labelling, with a mean of 95.3 ± 2.2 reflecting the ability of flow cytometry to detect collagen VI both intracellularly and on the cell surface (Fig. 2a). To assess intracellular accumulation, these data were expressed as a difference between permeabilised cells and non-permeabilised cells (Fig. 2b).

Patient groups were compared to control groups. The non-permeabilised cells demonstrated a marked reduction of collagen VI surface expression (19.6 ± 10.0) in the UCMD group compared to the control group (65.3 ± 10.7), with more than 1/3 reduction of collagen VI expression (*p* < 0.001). The BM group showed a mild decrease of collagen VI expression (54.1 ± 8.0) compared to the controls, which, however, was not statistically significant (*p* < 0.1) (Fig. 2a).

When cells were permeabilised prior to staining, over 90% of cells were positive for collagen VI expression in both patient groups as well as the control group. This is in keeping with previous observations that in most UCMD and in some BM fibroblasts, the reduction in extracellular collagen VI is accompanied by marked intracellular accumulation of mutated collagen VI molecules [1,21].

Using the difference between permeabilised and non-permeabilised cells, we noted a marked increase in collagen VI intracellular accumulation in UCMD fibroblasts (70.9 ± 7.5%) relative to control fibroblasts (30 ± 10.3%), which was statistically significant (*p* < 0.001). This confirms previous findings in UCMD using standard immunocytochemical methods, which revealed reduced collagen VI expression in the extracellular matrix in non-permeabilised cells and the intracellular accumulation of collagen VI in permeabilised cells [1]. We then analysed the difference between permeabilised and non-permeabilised cells in the BM group and found evidence of mildly increased intracellular accumulation of collagen VI (42.0 ± 8.5%) which when compared to controls was statistically significant (*p* < 0.05) (Fig. 2b).

Out of a total of 13 patient samples, all eight UCMD cases showed clear reductions in collagen VI expression in fibroblasts with non-permeabilised cell collagen VI expression ranging 9.7–36.8%, compared to control samples, which ranged between 50.0% and 76.9%. Among the five BM cases, the reduction of collagen VI expression was rather mild (44.8–66.2%) when compared to controls (50.0–76.9%). Flow cytometry results of non-permeabilised cells and differences between permeabilised and non-permeabilised cells demonstrated decreased collagen VI expression with increased levels of intracellular collagen VI accumulation in UCMD and BM patients compared to controls in all but one of the patients sampled (BM3).

## 4 Discussion

UCMD and BM are common forms of muscular dystrophies in childhood and, indeed, are the most common congenital muscular dystrophy variants in the UK population (FM, personal observation). While the combination of their characteristic clinical features and muscle imaging findings often allows us to suspect these conditions, the diagnostic pathway leading to the confirmation of the underlying genetic defect is prolonged, expensive and complicated by the occurrence of both autosomal dominant and autosomal recessive inheritance in each of the three collagen VI genes (*COL6A1*, *COL6A2* and *COL6A3*). Further complicating this pathway is the high residual level of collagen VI produced in BM. Arriving at a final definition of UCMD and BM at the genetic level is nevertheless necessary to rule out other similar conditions, provide accurate genetic counselling to families and will be necessary to recruit patients in future clinical trials. A prerequisite for the genetic analysis of the 107 exons of the *COL6A1*, *COL6A2* and *COL6A3* genes is the demonstration of abnormal collagen VI protein expression. While this is straightforward in patients with UCMD, in whom muscle biopsy often identifies an abnormality, the biochemical diagnostic process is complicated for those patients who fall at the mild end of the collagen VI myopathy phenotypic spectrum with Bethlem myopathy. Indeed, dual immunofluorescence labelling of muscle biopsies (for collagen VI and perlecan) is often apparently normal in BM patients. Immunofluorescence (IF) labelling of collagen VI in skin fibroblasts can demonstrate abnormalities in BM, and in some series 78% of genetically confirmed BM patients were identified in this way [21]. This technique, however, is labour-intensive, and the interpretation of borderline IF labelling results subjective. In our experience, a significant proportion of BM patients show immunofluorescence labelling of fibroblasts which is either normal or only subtly increased intracellularly, which is not a specific finding [1]. A more reproducible and more rapid method for detecting the abnormal collagen VI expression in UCMD and BM would not only help to select patients for subsequent molecular genetic analysis but could also help to assign the pathogenic significance of molecular variants in the collagen VI genes.

Flow cytometry analysis is both a more sensitive and a quantitative technique which has never been used before for assaying collagen VI expression. In the present study we performed flow cytometry analysis and compared the results with those results obtained from conventional immunofluorescent labelling of fibroblasts, using the same collagen VI antibody and the same fibroblast cell cultures. Although this study was limited to 13 patients with genetically confirmed collagen VI myopathies, flow cytometry analysis rapidly and clearly detected collagen VI deficiency in all eight UCMD patients. These results suggest that flow cytometry analysis is highly sensitive in detecting severe collagen VI deficiency, similar to what has been previously demonstrated using conventional immunohistochemistry [1,20,22]. In addition to conventional studies, flow cytometry allowed us to precisely quantify the deficiency of collagen VI in the UCMD patients analysed as less than 1/3 of the levels of collagen VI expressed in control fibroblast samples.

In some BM patients flow cytometry analysis detected a reduction of collagen VI expression. This was not a consistent finding, however, which accounts for the non-statistical significance of this testing in the BM group of patients. The comparison of collagen VI expression in individual Bethlem myopathy patients (non-permeabilised cells) compared to controls demonstrated that three of the five BM patients had clearly reduced collagen VI protein expression levels (BM2 with 51.3%, BM4 with 51.3% and BM5 with 44.8%) and one (BM1 with 56.9%) had moderately reduced collagen VI protein expression compared to our controls. One BM patient’s collagen VI expression, however, was slightly greater than the controls (BM3 with 66.2% versus control average 65.3%). It is interesting to note that patients with the same collagen VI mutation, for instance, patients BM4 and BM5 (father and son, respectively, with the same *COL6A1* heterozygous mutation) demonstrated comparable flow cytometry results (Table 1), once again confirming the robustness of this technical approach.

As flow cytometry analysis offers the ability to quantify collagen VI expression, we looked to see if clinical severity correlated with collagen VI expression level. When comparing the two diagnostic categories, there was a very good discrimination of patients in the UCMD category with all flow cytometry results (non-permeabilised cells) below 37% and all BM patients with flow cytometry results above 44%. These results suggest that flow cytometry analysis could be a useful tool when assigning disease severity in young infants with collagen VI myopathies, before the acquisition of motor milestones which may allow a confident clinical diagnosis to be made. It is nevertheless important to place any data obtained with laboratory techniques within a clinical context.

In this study we demonstrate that flow cytometry analysis can reliably quantify the deficiency of collagen VI observed in UCMD patients. The results in BM patients were more variable and reflect the higher level of collagen VI protein expression in these patients. Using non-permeabilised cells in the BM group there was evidence of a mild reduction of collagen VI expression when compared to controls, which was not statistically significant. In individual BM patients, however, collagen VI expression values were in the lower range of what was observed in control cells, suggesting that in a proportion of BM patients flow cytometry analysis may not be able to detect a sufficient difference in collagen VI expression when compared to control samples. This is not surprising, as mild BM patients can have predominantly qualitative differences in collagen VI network organisation [21], which are not assessed by either the standard immunohistochemical staining technique or the flow cytometry analysis technique. Therefore, care should be used when interpreting a flow cytometry result which does not demonstrate clearly decreased collagen VI expression in patients with suspected BM. In these patients additional studies on fibroblast immunolabelling techniques focused on the assessment of subtle quantitative and qualitative differences in collagen VI expression, might be required, although they also have shortcomings such as subjectivity of interpretation and lack of specificity of abnormalities observed. Other studies including muscle magnetic resonance imaging (MRI) could be incorporated into the diagnostic repertoire in this group of patients, as this imagining modality has been reported to detect disease-specific imaging patterns in relation to genetic diagnosis with a sensitivity of 90% and a specificity of 96% in a cohort of patients with muscular dystrophy and spinal rigidity [23–25]. This analysis, however, requires the collaboration of children, or the use of a general anaesthetic in children below the age of 5 years.

Our results suggest that flow cytometry analysis could represent a useful technique for the rapid, quantitative assessment of collagen VI expression in skin-derived fibroblasts. In this capacity, flow cytometry analysis could serve as a screening test for collagen VI deficiency both to identify individuals in whom to initiate the molecular genetic analysis or as a means to help assign pathogenicity of genetic variants of unknown significance.

## Figures and Tables

**Fig. 1a:**
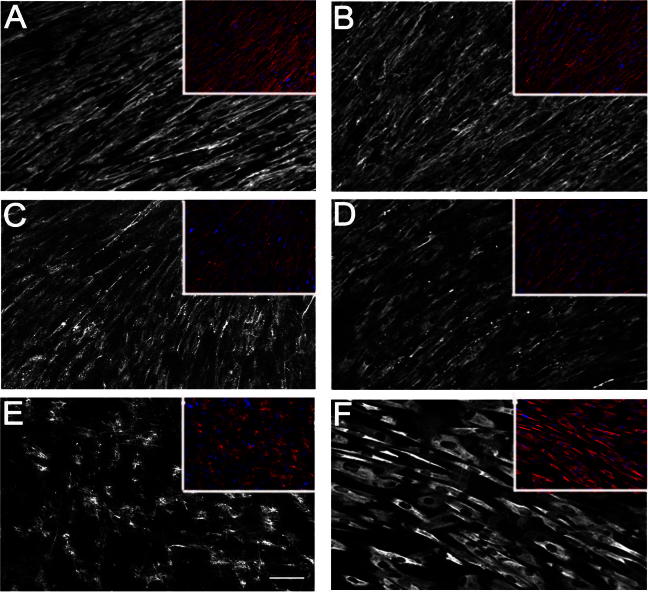
Collagen VI production from fibroblasts. Collagen VI immunolabelling of fibroblast cultures illustrating the different patterns of collagen VI abnormalities. Fibroblasts were labelled with an antibody to collagen VI without permeabilisation (A, C and E) and with permeabilisation (B, D and F). Control fibroblasts (A and B) showed the normal extracellular network of collagen VI microfibrils with/without permeabilisation with no intracellular accumulation when cells were permeabilised. Fibroblasts from a BM patient (BM4) showed a degree of reduction in collagen VI expression (C) with moderate intracellular labelling in some cells following permeabilisation (D). Fibroblasts from a UCMD patient (UCMD7) showed significant reduction of collagen VI expression in the ECM (E). When cells were permeabilised (F) marked intracellular accumulation was seen in the vast majority of cells. Nuclei were stained with Hoechst to control for cell density. (Scale bar = 25 μm).

**Fig. 1b:**
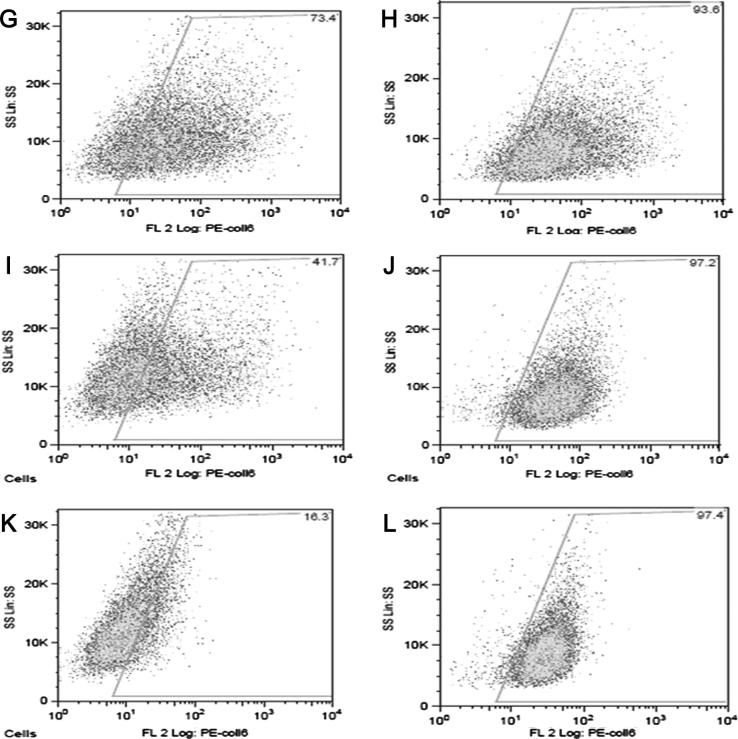
Flow cytometry analysis. Flow cytometry analysis was performed in order to compare the outcome of two methods with the same fibroblasts in each category shown as (G–L) (G, I and K indicates without permeabilisation and H, J and L indicates following permeabilisation). A Total of 15,000 cells were analysed and gated using the side scatter on the *y*-axis and fluorescence intensity of collagen VI immunolabelling (PE-collagen VI) on the *x*-axis. Immunolabelling of collagen VI positive gate was set up by negative control (where primary antibody is omitted). Control (G) showed approximately 73% of non-permeabilised cells were positively labelled with collagen VI in ECM. While BM4 (I) showed mild collagen VI reduction (approximately 42%), UCMD7 (K) showed severely decreased collagen VI expression in non-permeabilised cells (approximately 16%).

**Fig. 2a:**
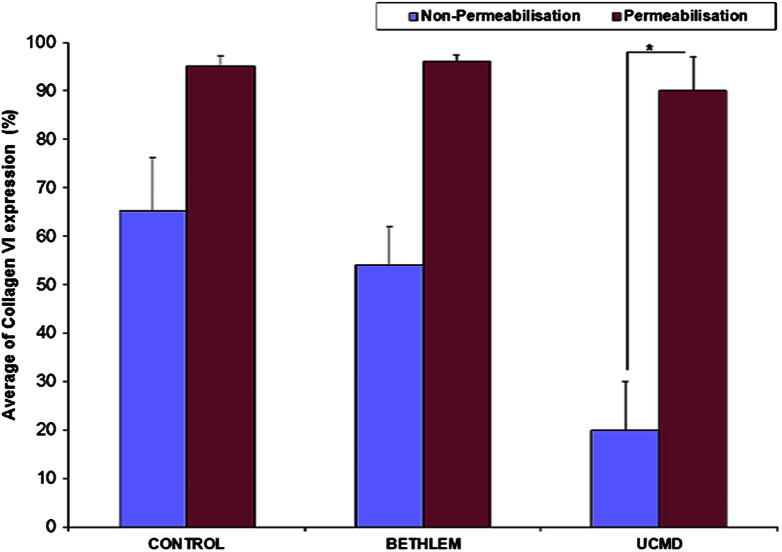
Quantification of collagen VI expression in fibroblasts determined by flow cytometry. Patient groups were compared to control group with/without permeabilisation. Collagen VI expression in non-permeabilised cells was the highest in the control group (just below 65%) compared to approximately 54% in the BM group and approximately 20% in the UCMD group. (^∗^=statistically significant; *p*<0.001).

**Fig. 2b:**
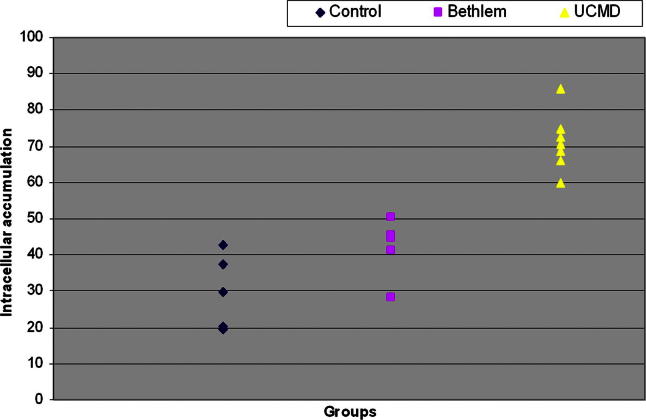
Quantification of collagen VI intracellular retention as determined by flow cytometry. The difference between permeabilised and non-permeabilised cells was used to determine the level of intracellular retention of collagen VI. Collagen VI retention was definitively higher in the UCMD group compared to the BM and control groups (*p* < 0.001). The BM group demonstrated less intracellular retention than the UCMD group; however, the levels of retention did not always exceed control group levels (*p* < 0.05).

**Table 1:** Clinical features and collagen VI molecular and biochemical status.

Patient	BM1	BM2	BM3	BM4	BM5	UCMD6	UCMD7
Current age (years)	47	39	†	42	7	16	21

Presentation	Birth: hip dislocation	2 years	Birth: hip dislocation	Infancy	Infancy: hypotonia and torticollis	Infancy: muscle weakness and progressive joint contractures	Birth: hip dislocation

Contractures	Elbows: + Knee: ++	Elbows: +++	Elbows: +++	Elbows:+++ Long finger flexors: +++ Achilles tendons: +++	None	Elbows: +++ Hips: +++ Knees: +++ Achilles tendons: +++	Elbows:+++ Hips:+++ Knees:+++

Maximum motor ability	Independent ambulation	Independent ambulation	Independent ambulation	Independent ambulation. Using walking frame since age 40 years	Independent ambulation	Independent ambulation. Wheelchair dependence since age 3.5 years	Independent ambulation. Wheelchair dependence since age 10 years

Respiratory function: % predicted forced vital capacity (FVC)	Not available	Not available	FVC 70% (age 23 years)	Not available	FVC 80% (age 6 years)	FVC 15% (age 9 years)	FVC 40% (age 12 years)

Mutation	*COL6A1*: intron 3 G > A + 1, causing the activation of a cryptic donor splice site in exon 3, resulting in an in-frame 66 nucleotide deletion	Heterozygous Gly341Val in exon 14 of *COL6A1*	Heterozygous mutation resulting in skipping of exon 14 of *COL6A1*	Heterozygous c.877G > A; p.Gly293Arg in exon 10 of *COL6A1*	Heterozygous c.877G > A; p.Gly293Arg in exon 10 of *COL6A1*	Heterozygous c.6210 + 1G > A in intron 16 of *COL6A3*	Homozygous c.1776 + 1G > A in intron 27 of *COL6A1*

Muscle collagen VI double immuno-labelling	Muscle not available	Muscle not available	Reduced at the sarcolemma	Muscle not available	Muscle not available	Reduced at the sarcolemma	Muscle not available

Fibroblast collagen VI immuno-labelling	Moderate reduction in the ECM; increased intracellular accumulation following permeabilisation. Overall collagen VI reduction: ∗∗	Moderate reduction in the ECM; increased intracellular accumulation following permeabilisation. Overall collagen VI reduction: ∗∗	Moderate reduction in the ECM; increased intracellular accumulation following permeabilisation. Overall collagen VI reduction: ∗∗	Moderate reduction in the ECM; increased intracellular accumulation following permeabilisation. Overall collagen VI reduction: ∗∗	Marked reduction in the ECM; marked intracellular accumulation following permeabilisation. Overall collagen VI reduction:∗∗∗	Marked reduction in the ECM; marked intracellular accumulation following permeabilisation. Overall collagen VI reduction:∗∗∗	Marked reduction in the ECM; marked intracellular accumulation following permeabilisation. Overall collagen VI reduction:∗∗∗

Flow cytometry result•	41.2%	45.6%	28.1%	44.6%	50.5%	70.7%	60.0%

References	[26,27]					[1,8,9]	[1,8]


Patient	UCMD8	UCMD9	UCMD10	UCMD11	UCMD12	UCMD13
Current age (years)	19	15	30	8	23	9

Presentation	1.5 years	Birth: hip dislocation and hypotonia	1 year	1 year	Birth: talipes	Birth: talipes

Contractures	Elbows: ++ Long finger flexors: + Knees: + Achilles tendons: +++	Elbows: ++ Hips: +++ Knees: +++	Elbows:+++ Hips: ++ Knees: + Achilles tendons: +++	Elbows: ++ Knees: +	Elbows: ++ Achilles tendons: ++	Elbows: ++ Hips: + Knees: ++ Achilles tendons: ++

Maximum motor ability	Independent ambulation. Wheelchair dependence since age 11 years	Independent ambulation. Wheelchair dependence since 6 years of age	Independent ambulation. Wheelchair dependence since age 15 years	Independent ambulation. Wheelchair dependence since age 8 years	Independent ambulation. Part-time wheelchair use since age 18 years	Independent ambulation. Wheelchair dependence since age 8 years

Respiratory function: % predicted forced vital capacity (FVC)	FVC 8% (age 15 years)	FVC 16% (age 12 years)	FVC 17% (age 19 years)	Not performed	FVC 38% (age 18 years)	FVC 56% (age 7 years)

Mutation	Homozygous Cys777Arg in exon 26 of *COL6A2*	Heterozygous donor splice site change in intron 16 of *COL6A3*	Homozygous c.2839_2850del; p.Leu947_Gly950del in exon 28 of *COL6A2*	Homozygous mutation r.2839_2850del; p.Leu947Gly950del detected in exon 28 of the *COL6A2* gene	Heterozygous mutation r.868G>A; p.Gly290Arg detected in exon 10 of the *COL6A1* gene	Heterozygous mutation r.868G>C; p.Gly290Arg detected in exon 10 of the *COL6A1* gene

Muscle collagen VI double immuno-labelling	Muscle not available	Muscle not available	Reduced at the sarcolemma	Reduced at the sarcolemma	Muscle not available	Reduced at the sarcolemma

Fibroblast collagen VI immuno-labelling	Marked reduction in the ECM; marked intracellular accumulation following permeabilisation. Overall collagen VI reduction: ∗∗∗	Marked reduction in the ECM; marked intracellular accumulation following permeabilisation. Overall collagen VI reduction: ∗∗∗	Marked reduction in the ECM; marked intracellular accumulation following permeabilisation. Overall collagen VI reduction: ∗∗∗	Marked reduction in the ECM; marked intracellular accumulation following permeabilisation. Overall collagen VI reduction: ∗∗∗	Marked reduction in the ECM; marked intracellular accumulation following permeabilisation. Overall collagen VI reduction: ∗∗∗	Marked reduction in the ECM; marked intracellular accumulation following permeabilisation. Overall collagen VI reduction: ∗∗∗

Flow cytometry result•	68.6%	65.9%	85.8%	72.5%	74.9%	68.8%

References	[1,8]	[1,8,9]	[1,20]		[1,20]	

Flow cytometry results for controls (difference between permeabilised cells and non-permeabilised cells): 19.6–42.9%. ECM = extracellular matrix. + = mild contractures (5°–10°). ++ = moderate contractures (>10° but <30°). +++ = severe contractures (>30°). ∗ = mild collagen VI reduction in ECM. ∗∗ = moderate collagen VI reduction in ECM. ∗∗∗ = marked collagen VI reduction in ECM.

† Deceased (died of lung cancer at age 30 years).

• Difference between permeabilised cells and non-permeabilised cells (= intracellular accumulation).
